# 1.8 Å resolution structure of β-galactosidase with a 200 kV CRYO ARM electron microscope

**DOI:** 10.1107/S2052252520006855

**Published:** 2020-06-11

**Authors:** Alan Merk, Takuma Fukumura, Xing Zhu, Joseph E. Darling, Reinhard Grisshammer, Jana Ognjenovic, Sriram Subramaniam

**Affiliations:** a Cancer Research Technology Program, Frederick National Laboratory for Cancer Research, Leidos Biomedical Research, Inc., Frederick, MD 21701, USA; b JEOL Inc, Tokyo, Japan; c University of British Columbia, Vancouver, British Columbia V6T 1Z3, Canada; d National Cancer Institute Frederick Office of Scientific Operations, Frederick, MD 21701, USA

**Keywords:** cryo-EM, beta-galactosidase, cold field emission gun, 200 kV, *SerialEM*

## Abstract

We report the determination of the structure of *Escherichia coli* β-galactosidase at a resolution of ∼1.8 Å using data collected on a 200 kV CRYO ARM microscope equipped with a K3 direct electron detector. The data were collected in a single 24 h session by recording images from an array of 7 × 7 holes at each stage position using the automated data collection program *SerialEM*. In addition to the expected features such as holes in the densities of aromatic residues, the map also shows density bumps corresponding to the locations of hydrogen atoms. The hydrogen densities are useful in assigning absolute orientations for residues such as glutamine or asparagine by removing the uncertainty in the fitting of the amide groups, and are likely to be especially relevant in the context of structure-guided drug design. These findings validate the use of electron microscopes operating at 200 kV for imaging protein complexes at atomic resolution using cryo-EM.

## Abstract 2

An electron microscope operating at a voltage of 200 kV is shown to be sufficient for determination of protein structures at high resolution using cryo-EM methods. The level of detail in the 1.8 Å map reported by the authors shows visible density bumps for hydrogen atoms and marks an important advance in the establishment of microscopes operating at lower voltages as powerful tools for structure-guided drug design using cryo-EM.

## 1. Introduction   

As of April 2020, there were twelve entries in the Electron Microscopy Data Bank for structures reported at resolutions better than 2.0 Å using single-particle cryo-electron microscopy (cryo-EM) (https://www.ebi.ac.uk/pdbe/emdb). Eight of these are structures (including one unpublished entry) of apoferritin (Danev *et al.*, 2019 ▸; Hamaguchi *et al.*, 2019 ▸; Kato *et al.*, 2019 ▸; Wu *et al.*, 2020 ▸), two are of β-galactosidase (Bartesaghi *et al.*, 2018 ▸; Zivanov *et al.*, 2018 ▸) determined from the same data set (Bartesaghi *et al.*, 2015 ▸) and one each from bovine glutamate de­hydrogenase and the adeno-associated virus (AAV) capsid (Merk *et al.*, 2016 ▸; Tan *et al.*, 2018 ▸). With the exception of one of the entries for apoferritin that was collected on a 200 kV Thermo Fisher Talos Arctica microscope, the rest are structures determined from data collected using electron microscopes operated at 300 kV.

Electron microscopes that operate at a maximum voltage of 200 kV can be significantly cheaper and have room-height requirements that are met more easily and at a lower cost than those designed to operate at 300 kV. Given the pressing need in the structural biology community for lower cost instrumentation that is still capable of delivering high-resolution cryo-EM structures, we have evaluated the potential of a JEOL 200 kV CRYO ARM microscope for atomic resolution cryo-EM using the protein β-galactosidase as a benchmark (https://www.ebi.ac.uk/pdbe/emdb/empiar/reuse).

## 2. Methods   

*E. coli* β-galactosidase was purchased (Sigma–Aldrich, catalog No. G5635), dissolved in a buffer comprising 25 m*M* Tris–HCl (pH = 8), 50 m*M* NaCl, 0.5 m*M* Tris­(2-carb­oxy­ethyl)­phosphine (TCEP) and 2 m*M* MgCl_2_ (Bartesaghi *et al.*, 2014 ▸), and subjected to size-exclusion chromatography using a Superose 6 Increase 10/300GL column (GE Healthcare, catalog No. 29091596) equilibrated with the same buffer at a flow rate of 0.5 ml min^−1^. The resulting peak β-galactosidase protein fraction at 4.5 mg ml^−1^ was deposited onto freshly plasma-cleaned (Solarus apparatus from Gatan, Inc. Pleasanton, CA) 200 mesh Quantifoil R1.2/1.3 grids (Quantifoil Micro Tools GmbH, Jena, Germany), and the grids were plunge-frozen using a Leica EM GP instrument (Leica Microsystems Inc., Buffalo Grove, IL, USA).

Data were collected on a CRYO ARM 200 microscope (JEOL Inc. Tokyo, Japan) equipped with a cold field emission gun and an in-column omega filter which are standard features on this instrument. We used a 30 eV slit width and collected images on a K3 direct electron detector (Gatan Inc.). All images were recorded with *SerialEM* software using a 7 × 7 hole pattern and active beam-tilt compensation applied. Target hole centering and autofocusing were performed via stage movement. After the stage position was set, drift was measured on an adjacent carbon area and data collection imaging was delayed until the measured drift fell below 2 Å s^−1^. All images were acquired in super-resolution mode at a nominal magnification of 100 000×, corresponding to a pixel size of 0.26 Å on the specimen, over a target defocus range from −0.8 to −0.95 µm. Each image was fractionated into 40 frames with a frame exposure of 0.025 s, at a dose rate of ∼11 e^−^ physical pixel^−1^ s^−1^, to accumulate a total dose of ∼40 e^−^ Å^−2^. Cold FEG flashing was performed automatically by *SerialEM* every 4 h.

A total of 4949 movies were collected. All image processing was performed in the open-source program *RELION* (v. 3.1-beta). Using the wrappers in *RELION*, movie frames were aligned with *MotionCor2* and the contrast transfer function parameters for each micrograph were estimated with *CTFFIND* (v. 4.1.13). Autopicking using LoG picker in *RELION* yielded 998 945 particles, which were subjected to 2D classification to remove junk particles. The remaining 531 649 particles were subjected to 3D classification, which separated the particles into four high-resolution classes and two low-resolution classes. The 257 202 particles from the high-resolution classes were put into auto-refinement, which resulted in a 2.7 Å resolution structure. An initial round of CTF refinement correcting only for per-particle defocus, per-micrograph astigmatism and beam tilt, improved the resolution to 2.4 Å. A second round of CTF refinement correcting for higher-order aberrations and enlarging the box size improved the resolution to 2.0 Å. Bayesian polishing further improved the resolution to 1.9 Å. Additional rounds of CTF refinement and Bayesian polishing ultimately resulted in a final resolution of 1.82 Å (using the same mask as that used for the resolution estimation of EMD-0153). The procedures we followed for post-processing were standard procedures recommended by the developers of *RELION*, with global *B*-factor correction based on Guinier fitting. Model building and refinement were performed in *Coot* (v. 0.8.9.2) and *PHENIX* (v. 1.17.1), respectively (model statistics are given in Table 1 ▸).

## 3. Results and discussion   

We recorded a set of 4949 movies in a single 24 h session from a plunge-frozen specimen of β-galactosidase using a JEOL 200 kV CRYO ARM microscope equipped with a Gatan K3 direct electron detector. The data were collected and processed using the open source software programs *SerialEM* and *RELION*, respectively (see *Methods* [Sec sec2] for details). The final resolution of the map, as assessed by the conventional Fourier shell correlation (FSC) criterion, is 1.8 Å, as judged by both the FSC value at 0.143 between random halves of the data, and the FSC value at 0.5 between the map and the atomic model derived from the map (Fig. S1 of the supporting information). The final atomic model has 3618 water molecules, closely comparable to the number of water molecules reported in high-resolution crystal structures of β-galactosidase (Juers *et al.*, 2003 ▸, 2000 ▸).

**Figure 1:**
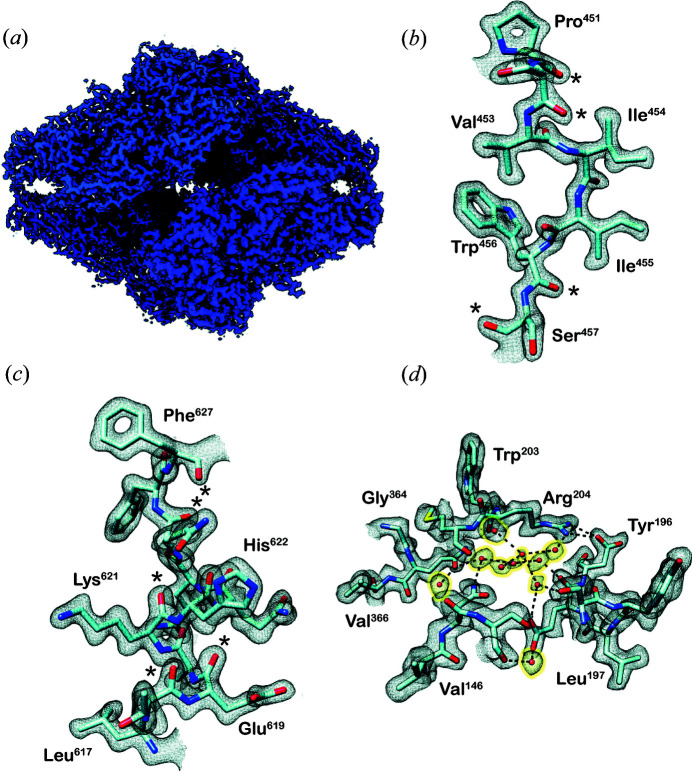
Cryo-EM density map of β-galactosidase at 1.8 Å resolution. (*a*). Surface representation of the β-galactosidase tetramer. (*b*)–(*d*) Visualization of selected map regions showing delineation of secondary-structural elements and amino-acid densities. To provide a direct comparison with our earlier work, the regions shown here are identical to selected panels of Figs. 1 and 2 from our previous work (Bartesaghi *et al.*, 2018 ▸), where we reported a 2.2 Å map of β-galactosidase using data collected on a Titan Krios electron microscope operated at 300 kV. The segments display ‘holes’ in residues such as (*b*) the pyrrolidine ring in Pro451 and the indole group of Trp456, and (*c*) in Phe627 with the side-chain densities delineated more clearly than in the earlier 2.2 Å map. The region of the protein highlighted in panel (*d*) shows three additional water molecules to that reported in the corresponding panel in the 2.2 Å map [Fig. 2(*a*) in our previous work (Bartesaghi *et al.*, 2018 ▸)].

**Figure 2:**
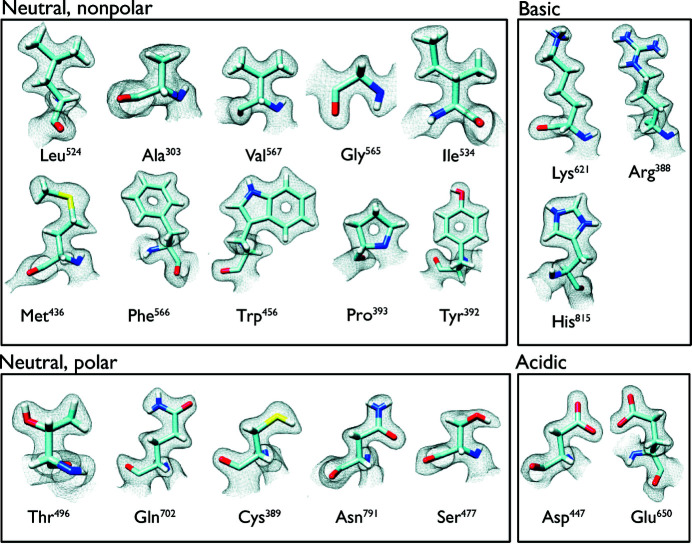
Illustration of map quality for each type of amino-acid side chain. The individual panels show the visualization of map density for representative examples of each of the 20 standard amino acids, which are grouped into neutral (nonpolar and polar), basic and acidic categories. Density contribution from hydrogens is visible in the side chains.

To assess the quality of the map obtained from the CRYO ARM 200 microscope, we show in Fig. 1 ▸ panels from the same map regions that we reported in 2015 for the 2.2 Å cryo-EM structure of β-galactosidase obtained using a Titan Krios 300 kV microscope (Bartesaghi *et al.*, 2015 ▸). Visual inspection of the map for features such as delineation of the side chains, holes at the center of residues such as Phe and Pro, as well as densities for water molecules demonstrate that the map quality is better than what we have previously reported for β-galactosidase at resolutions of 2.2 Å (Bartesaghi *et al.*, 2015 ▸) and 1.9 Å (Bartesaghi *et al.*, 2018 ▸) with data collected at 300 kV. To quantitatively compare our present data with the earlier 1.9 Å resolution Krios data, we computed plots of the increase in map resolution with the amount of data using approaches suggested by Rosenthal & Henderson (2003 ▸) and by Stagg *et al.* (2014 ▸) (Fig. S2). The fitted lines through the points plotted suggest that resolutions below 1.9 Å can be obtained with fewer particles on the CRYO ARM 200, whereas the amount of data needed to reach 1.9 Å is similar for the Krios and the CRYO ARM 200. As another metric, we present a panel of 20 amino acids that we and others have typically used in the past to evaluate map quality in high-resolution structures [compare Fig. 2 ▸ with Figure 4 in our previous work (Bartesaghi *et al.*, 2015 ▸)]. The individual side-chain densities in our current map compare favorably with atomic resolution X-ray structures.

**Figure 3:**
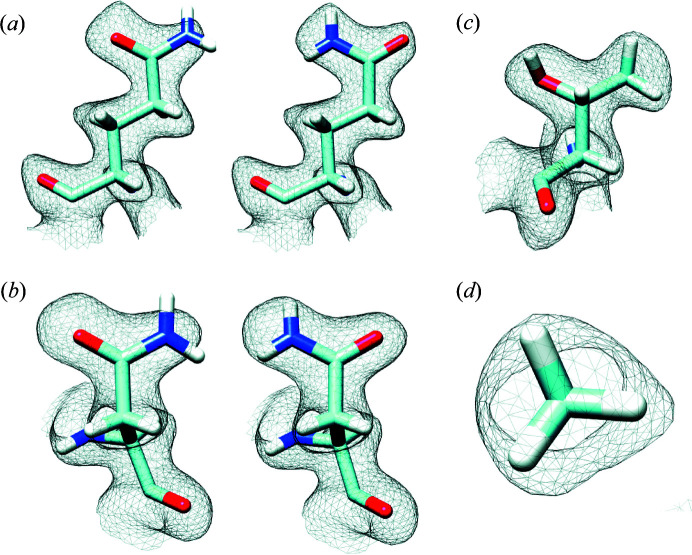
Density bumps from hydrogen atoms enable correct rotamer assignment. Densities for the hydrogens emanating from the nitro­gen atoms of (*a*) glutamines and (*b*) asparagines enable discrimination between incorrect (left) and correct (right) orientations of the amide group in the density. The density for the three hydrogen atoms in Thr441 visualized in (*c*) side and (*d*) end-on views allows assignment of the rotamer of threonines because of the triangular shape of the projection viewed perpendicular to the plane of the three hydrogen atoms.

A striking feature of the map is that the densities for individual amino-acid side chains in most cases display density bumps corresponding to the locations of hydrogen atoms (Fig. 2 ▸), a feature that was first reported in the map of the AAV capsid at 1.86 Å resolution (Tan *et al.*, 2018 ▸). Although the map is not yet at a resolution where individual hydrogen atoms are separately resolved, these additional density bumps nevertheless allow for more accurate model building. This can be demonstrated most clearly for the cases of glutamine and asparagine residues, where the hydrogen density removes the uncertainty in the fitting of the amide groups. This is because the asymmetric density distribution [see Figs. 3 ▸(*a*) and 3 ▸(*b*)] allows unambiguous discrimination of the correct orientation. For threonine residues such as Thr441, the density contribution from hydrogen atoms allows clear distinction between the density for the gamma carbon and that for the gamma oxygen [Figs. 3 ▸(*c*) and 3(*d*)], also enabling correct rotamer selection. It has been suggested that as many as 20% of Asn/Gln rotamers in X-ray structures may be incorrect (Weichenberger & Sippl, 2006 ▸), implying a unique role for high-resolution cryo-EM density maps in this context. Because of the density for the hydrogen branching from the β-carbons of the valine and threonine residues, in some instances it can be challenging to ascertain whether the density near the β-carbon corresponds to the hydrogen atom or to the presence of an alternative side-chain conformation. Higher resolution is needed in order to eliminate these uncertainties and facilitate more automated, accurate model building. Some additional panels which highlight the map quality are displayed in Fig. S3.

The JEOL CRYO ARM 200 microscope has a cold field gun emitter and an embedded in-column energy filter as default features which are not available in 200 kV instruments marketed by Thermo Fisher (Talos Arctica and Glacios). We cannot yet ascertain whether these two additional features are necessary for obtaining optimal performance from the microscope, but the increased coherence of the cold field emission gun could be an important factor in obtaining higher resolution. The lenses in the filter are essentially part of the imaging system, so merely removing the slit will not provide an objective measure of the influence of having an in-column filter. Our expectation though is that imaging with and without the slit will probably produce similar results.

Recently, a 1.54 Å structure of apoferritin obtained using a JEOL CRYO ARM 300 microscope was reported (Kato *et al.*, 2019 ▸). Analysis of the data revealed a measurable change in beam tilt each time there was a large change in the objective lens setting. This was probably not a major limiting factor in this instance because the high symmetry of apoferritin enables beam tilt estimation to be achieved on a per-image basis. However, for particles with lower symmetry, it is often necessary to group together large numbers of images in order to be able to accurately estimate the beam tilt. Thus, any beam tilt changes could preclude high-resolution structure determination for this class of proteins. To avoid any potentially large beam tilt variations, we used stage movements to achieve the desired defocus for each exposure, thus allowing the objective lens setting to remain fixed throughout the data collection session.

Lander and colleagues first showed that resolutions better than 2 Å could be obtained from data collected at 200 kV using images of apoferritin acquired on a Thermo Fisher Talos Arctica microscope (Wu *et al.*, 2020 ▸). Our present report of a map for β-galactosidase at 1.8 Å resolution with the JEOL CRYO ARM 200 microscope shows that 200 kV instruments are adequate for high-resolution imaging of protein complexes with lower symmetry than apoferritin. Microscopes operating at lower voltages may allow for further cost reduction in instrumentation for high-resolution cryo-EM imaging (Naydenova *et al.*, 2019 ▸). Irrespective of the voltage used for imaging, the ability to directly visualize atomic level detail including hydrogen atoms will have an increasing impact on the relevance of cryo-EM in computational chemistry and computer-aided drug design. The map presented here obtained from imaging at 200 kV already shows tantalizing hints of resolving the geometry of water molecules, suggesting that it may soon be possible to determine the rotational register of the O—H bonds in structured water molecules, further enhancing the value of cryo-EM for precision drug design and modeling ligand binding.

The map and refined coordinates have been deposited in the Electron Microscopy Data Bank and the Protein Data Bank as entries EMD-21995 and 6x1q, respectively.

## Supplementary Material

Supporting figures. DOI: 10.1107/S2052252520006855/eh5008sup1.pdf

EMDB reference: β-galactosidase at ∼1.8 Å, EMD-21995

PDB reference: β-galactosidase at ∼1.8 Å, 6x1q

## Figures and Tables

**Table 1:** Data collection, processing, refinement and validation statistics

Grid type	Quantifoil 200 mesh Cu, R1.2/1.3
Plunge freezer	Leica EM GP
Microscope	CRYO ARM 200
Camera	K3
Slit Width (eV)	30
Nominal magnification (kx)	100
Physical pixel size (Å)	0.52
Dose rate (e^−^ pixel^−1^ s^−1^)	11
Total dose (e^−^ Å^−2^)	40
Micrographs	4949
Particles	257202
Symmetry imposed	*D*2
Average resolution (Å)	1.82
FSC threshold	0.143
Map sharpening *B* factor (Å^2^)	−26
	
Refinement	
Initial model used (PDB entry)	5a1a
Model resolution (Å)	1.8
FSC threshold	0.5
Model composition	
Atoms	66138
Hydrogen atoms	30520
Protein residues	4056
Waters	3618
*B* factors (Å^2^)	9.04
R.m.s. deviations	
Bond lengths (Å)	0.007
Bond angles (°)	0.716
Correlation coefficients	
Mask	0.87
Box	0.76
	
Validation	
*MolProbity* score	1.12
Clashscore	3.32
Poor rotamers (%)	0.36
Ramachandran plot	
Favored (%)	98.12
Allowed (%)	1.78
Disallowed (%)	0.10
