# Supplementary material for: 1.8 Å resolution structure of β-galactosidase with a 200 kV CRYO ARM electron microscope
Source: IUCrJ. 2020 Jun 11;7(Pt 4):639–43. doi: 10.1107/S2052252520006855 (PMC7340270; doi:10.1107/S2052252520006855)
Supplement: Supplementary file 1 [file m-07-00639-sup1.pdf]

IUCrJ

Volume 7 (2020)

Supporting information for article:

1.8 Å resolution structure of β-galactosidase with a 200 kV cryoARM electron microscope

Alan Merk, Takuma Fukumura, Xing Zhu, Joseph E. Darling, Reinhard Grisshammer, Jana Ognjenovic and Sriram Subramaniam

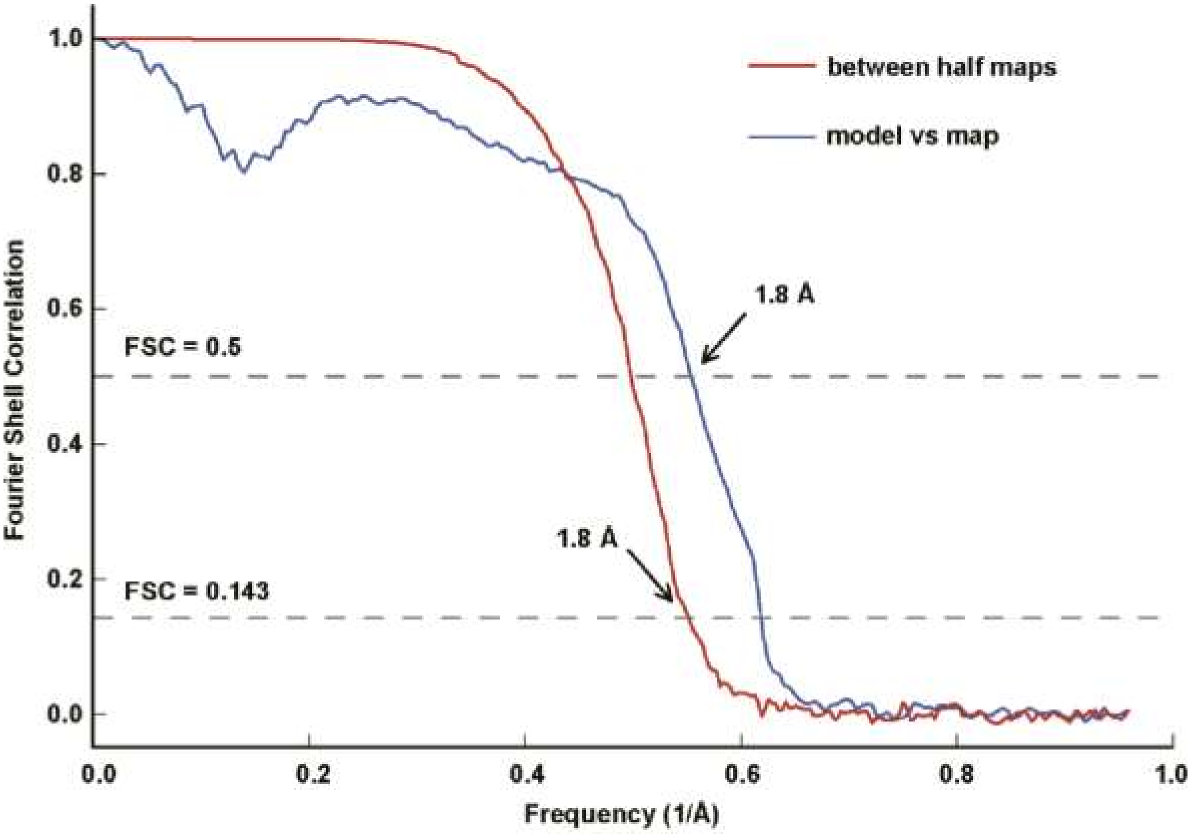

Figure S1 **Fourier Shell Correlation (**FSC) curves demonstrating an estimate of 1.8 Å resolution estimate. A. Gold-standard FSC between two independent half maps. B. FSC between the current map and its corresponding atomic model.

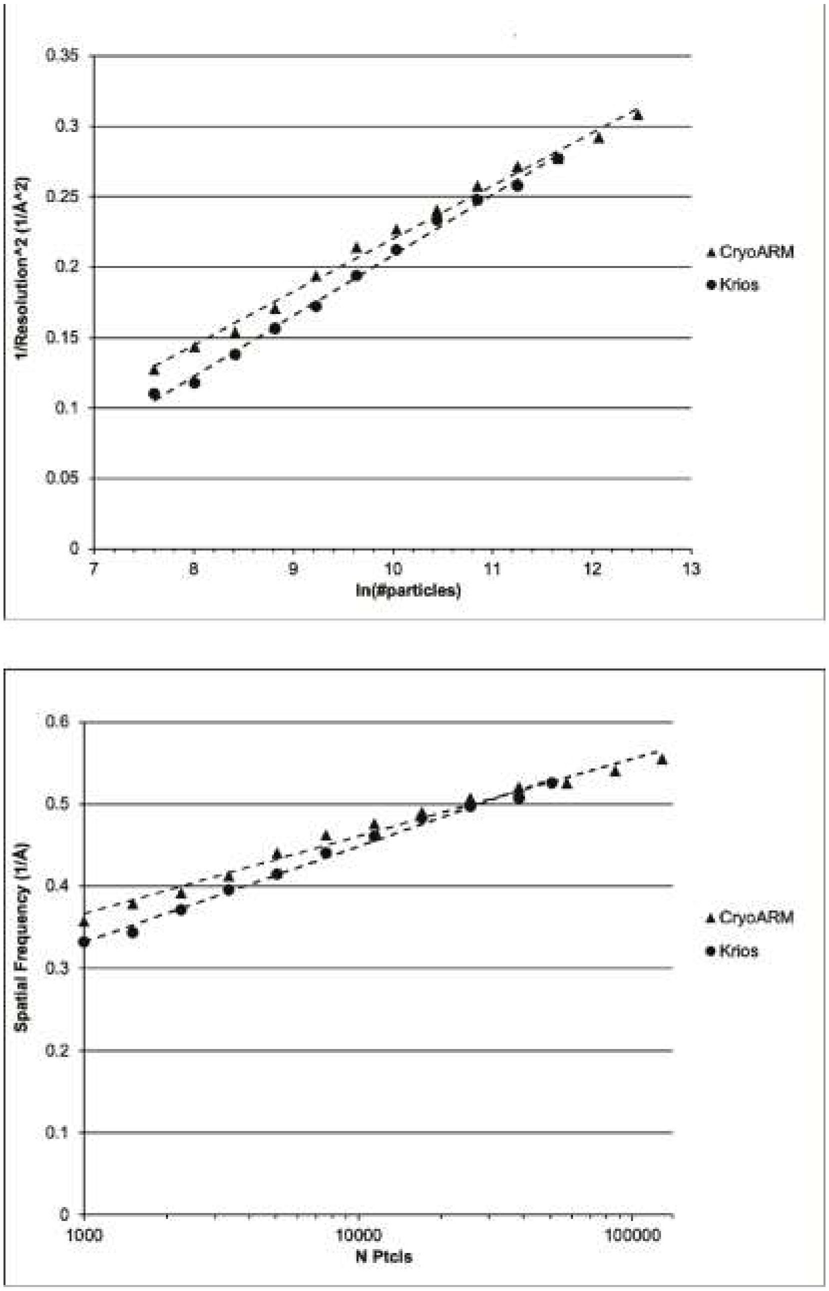

Figure S2 (A) B-factor plot (Rosenthal & Henderson, 2003). Square of 1/resolution is plotted against the logarithm of the number of particles in each refinement. (B) ResLog plot (Stagg *et al.*, 2014). Spatial frequency (1/resolution) of a reconstruction is plotted against the number of particles in half-sets of the refinement. Resolutions were estimated using the FSC 0.143 criterion.

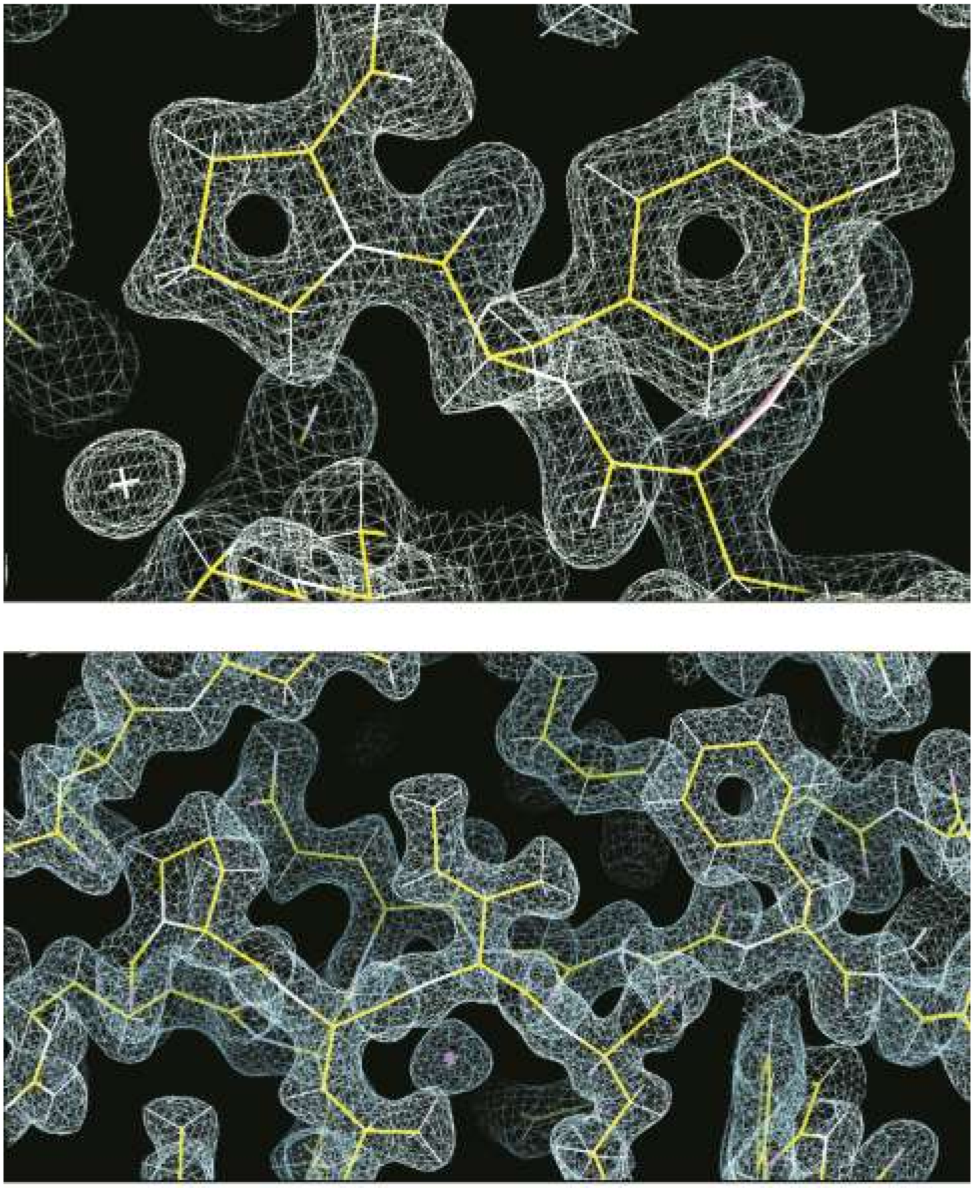

Figure S3 Visualization of selected regions of the density map in the COOT environment to highlight overall map quality.
